# The effectiveness and cost-effectiveness of ADHD care models: a systematic review

**DOI:** 10.1007/s00787-026-02959-y

**Published:** 2026-03-25

**Authors:** Russell Carter, Kaitlyn McKenna, Ha Le

**Affiliations:** 1 Faculty of Health, School of Health and Social Development Institution, Deakin University, Burwood, VIC Australia; 2 Deakin Health Economics, School of Health and Social Development, Institute for Health Transformation Faculty of Health, Deakin University, Geelong, VIC Australia

**Keywords:** Collaborative care, Integrated care, ADHD, Treatment models, Cost-effectiveness, Effectiveness

## Abstract

There are significant unmet needs in accessing healthcare services for Attention Deficit Hyperactivity Disorder (ADHD). A growing demand for ADHD assessment and treatment resulted in the necessity to implement effective care models and assess their effectiveness and cost-effectiveness to address the current substantial unmet needs of ADHD healthcare services. To explore and synthesise existing evidence of the effectiveness and cost-effectiveness of ADHD collaborative care models in a systematic review. Six databases were searched for peer-reviewed English publications via EBSCOhost. A two-stage screening process was conducted by two independent reviewers. Quality assessment was conducted using the National Heart, Lung, and Blood Institute Study Quality Assessment Tools. The search identified 2710 records, and 11 met the inclusion criteria. There were 7 models identified with varying levels of effective treatment approaches to ADHD. The mood, ADHD collaborative care, digital mental health intervention, and tele-mental health services models’ tele-health and ADHD treatment training for general practitioners (GP) improved ADHD treatment monitoring and reduced ADHD symptoms in children and adolescents. The nurse-led, multi-agency drop-in clinic, integrated care arrangements, and doctor office collaboration care models, consisting of mental health specialists and ADHD nurse specialists’ collaboration with GPs improved parents’ accessibility to ADHD resources. There were no cost-effectiveness studies of ADHD care models identified. The 7 ADHD care models demonstrated that collaborative and integrated care models increase accessibility to ADHD treatment, reduce ADHD symptoms and improve service delivery. Further research is needed to investigate the cost-effectiveness of these ADHD care models.

## Introduction and background

### ADHD definition and its health and social impacts

Attention Deficit Hyperactivity Disorder (ADHD) is a neurodevelopmental condition characterised by patterns of inattention, hyperactivity, and impulsivity that interfere with daily functioning and development [1–3]. It is commonly diagnosed in early childhood and adolescence [4, 5] and is one of the most common neurodevelopmental conditions, globally impacting about 5–7% of children and adolescents [6]. The exact cause of ADHD is not fully understood, but it is believed to involve a combination of genetic, neurological, and environmental factors. ADHD can accompany other neurodevelopmental conditions such as personality disorder and oppositional defiant disorder (ODD) [7]. Children and adolescents with ADHD often encounter many adverse effects in multiple areas such as exclusion from school [3], poor social skills [7], and reduced quality of life [5]. The risk of leaving ADHD untreated has immense and immediate economic and health impact, manifesting into challenges extending further into adulthood such as professional and social integration [3, 8]. Consequently, it is imperative that ADHD can be diagnosed in early childhood and adolescence to establish and implement appropriate treatment.

### ADHD diagnosis and treatment guidelines

Both the American Academy of Paediatrics (AAP) [8] and National Institute for Health and Care Excellence (NICE) [9] provide clinical practice guidelines advising the ADHD assessment/diagnosis process to involve a thorough comprehensive evaluation by a healthcare professional, including behavioural assessments and input from teachers or family members when necessary [2, 3]. The inclusion of parents and teachers is to collect evidence of ADHD symptoms [2] and ensure collective agreement of ADHD in multiple settings. In terms of ADHD treatment, a combination of prescribed medication and cognitive behaviour therapy are recommended [2, 3]. While these different forms of treatment have demonstrated effectiveness in treating children and adolescents with ADHD, however the adherence to the ADHD treatment guidelines set forth by AAP and NICE is not attainable for all children and adolescents diagnosed with ADHD for numerous reasons, such as a shortage of mental health workers [10], lack of accessible local resources [11], and limited mental health training of general practitioners [1, 10].

### ADHD treatment accessibility

The structural issues associated with accessibility to ADHD treatment have been well researched and documented [5, 12] however, there is a notable gap between the number of children diagnosed with ADHD and those receiving appropriate treatment [13]. For instance, the 2016 U.S. National Survey of Children’s Health revealed that while 8.4% of children were diagnosed with ADHD, nearly 23% of these children did not receive medication or cognitive behaviour therapy [13]. Currently, issues pertain to the adherence to ADHD treatment guidelines set by the AAP, including follow-up practices. Research [14] has shown that only 53% of general practitioners(GPs) who prescribed stimulant medications conducted routine follow-ups as recommended. Standardising treatment and ensuring regular monitoring of medication efficacy are critical for improving outcomes. However, coordination of communication among key stakeholders such as healthcare providers, families, and schools are often time-consuming, requiring additional work beyond the standard work responsibilities. The consequences of not diagnosing ADHD early on or leaving it untreated can increase the likelihood of developing other mental health issues such as anxiety, poor self-esteem, and depression [2, 12].

### ADHD treatment models

The severe gaps between the diagnosis and treatment of children and adolescents with ADHD requires comprehensive care models that both empower and enable key stakeholders to work cooperatively in addressing these disparities. There are a range of different ADHD care models within the healthcare system. For instance, one treatment model can consist of minimal interaction between GPs and mental health professionals where referrals are the main form of connection and communication may occur via phone or written correspondence, to fully integrated services in shared spaces, coordinate schedules, and share patient charts and care responsibilities [2, 3, 12]. The increasing recognition of ADHD as a prolonged condition [2] has led to the implementation and testing of ADHD collaborative care models guided by the Chronic Care Model (CCM) approach, utilised in GP settings [1, 10]. The CCM consists of a diverse care team, following evidence-based treatment approaches in the administering and monitoring of the defined patient population [10–12]. In a previous review published in 2018, [12] there were only six studies that explored the effectiveness of ADHD care models, which showed that methods of interdisciplinary coordination are an effective treatment approach. In fact, both systematic reviews [5, 12] have indicated that ADHD care models have improved accessibility of diagnosis and treatment for ADHD because of these models’ collaborative and integrative techniques. Therefore, the growing recognition of ADHD and its growing demand for treatment, requires a more contemporary synthesis of the new and current literature on the effectiveness and cost-effectiveness of current ADHD care models [12]. We aim to identify and synthesise the current existing literature on the effectiveness and cost-effectiveness of ADHD care models in the assessment and treatment of children with ADHD.

## Methodology

This systematic review was reported based on the PRISMA guidelines 2020 criteria [15] and was registered with the International Prospective Register of Systematic Reviews (CRD42024625482).

### Search strategy

The search strategy was developed by the primary reviewer (RC) in consultation with the review team and a librarian expert. The primary reviewer conducted the search (Appendix A, Table 1) in the following databases via EBSCOhost: APA PsycINFO, MEDLINE Complete, ERIC, and Health Policy Reference Centre, EconLit, and Global Health. Various search terms were used for each of the three main concepts of the search strategy: (1) ADHD, (2) Effectiveness/cost-effectiveness, (3) Care Models.

**Table 1:** ADHD Care ModelsAuthor, date

Aim		Study Design	Setting	Sample Characteristics	Model	Key Findings
Integrated Care Models						
Moore et al. [2]	To assess the impact of two co-located primary care models with varying levels of behavioural health integration.	Retrospective Cohort Analysis Non-experimental	Small to medium-sized GP practices in urban and suburban areas	6–13 years old (*n = 149)*	Integrated Care Arrangement	- Patients in fully integrated setting had better adherence to the American Academy of Paediatrics. - No reimbursable benefit of integrated care could reduce costs associated with poor medical follow-up. - Improvement in parent education on ADHD^1^ - Provided more advocacy for special education. - Fully integrated service had a higher level of behavioural health professional staffing compared to the co-located service. Once onsite contact with a Behavioural Health Practitioner for tasks like follow-up data, advocacy, and ADHD training was considered, practice differences became insignificant.
Chen et al. [8]	- To monitor and evaluate the implementation of integrated care arrangements in the treatment of ADHD by GPs^2^ in the paediatric centres. 1 General Practitioner	Retrospective Cohort Analysis Non-experimental	Small to medium-sized GP practices in urban and suburban areas	4–18 years old (*n = 4204)*	Integrated Care Arrangement	- Children with ADHD who were diagnosed by co-located and co-affiliated were almost twice as likely to begin treatment compared to those diagnosed by non-co-located GPs. - Compared to those diagnosed by non-co-located GPs, a higher treatment rate was noted in those diagnosed by co-located and co-affiliated GPs. - Only 7% of children with ADHD identified by GPs received psychotherapy as recommended by ADHD treatment guidelines.
Epstein et al. [1]	- To identify the sustainability outcomes by implementing ADHD guidelines within paediatric practices.	Longitudinal Observational Cohort	Small to medium sized GP practices in urban/suburban	Elementary school-aged *(n = 202)*	Quality Improvement	- GP retention and application of ADHD guidelines within paediatric practices demonstrates effectiveness in ADHD treatment training.
**Collaborative Care Models**						
Parkhurst et al. [10]	- To expand access to mental health services. -To provide ADHD treatment training to paediatricians.	Longitudinal Observational Cohort	Small to medium sized GP practices in urban/suburban	6–18 years old (*n = 149)*	Mood, Anxiety, ADHD Collaborative Care (MAACC)	- Implementation of model is costly and could not be established without philanthropic resources. - Digital monitoring tools were costly and ineffective. - Improvement in ADHD symptom monitoring by GPs. - Treatment team’s communication and collaboration supports existing evidence of model efficacy.
Parkhurst et al. [11]	- To extend the findings of collaborative care and provide supportive evidence for the program outcomes care.	Longitudinal Observational Cohort	Small GP clinics in regional areas	6–18 years old (*n = 208)*	MAACC	- Effective implementation of model is dependent on local resources. - Cannot be generalised for all regions (urban and suburban). - Parent-reported ADHD-inattentive symptoms improved from baseline to 6 and 18 weeks.
Lawrence-Sidebottom et al. [7]	- To determine the effects of a collaborative care on inattention, hyperactivity, oppositional symptoms.	Longitudinal Observational Cohort	Tele-Med Service	6–17 years old (*n = 107)*	DMHI^3^	- Significant improvements in a reduction of ADHD symptoms. - Duration of care was predicator for positive association of symptom change.
Albanna et al. [4]	-To investigate the effectiveness of tele-collaborative care for ADHD in primary health care centres.	RCT^4^	Tele-Med Service	6–12 years old (*n = 20)*	Collaborative Tele-Mental Health Services	- GPs were receptive to ADHD treatment training. - Waiting time for diagnosis and treatment was significantly shorter in the intervention cohort.
Kolko et al. [20]	To evaluate the outcomes of children diagnosed with comorbid ADHD.	RCT	Community GP Clinics	5–12 years old (*n = 206)*	DOCC^5^	- Collaborative care intervention incorporating ADHD treatment guidelines was more effective than psychoeducation and facilitated referral to community treatment for behaviour problems. - Limited provider availability and cost have hindered the implementation of these practices in paediatric primary care.
**Other Care Models**						
Sfar-Gandoura et al. [3]	To implement and evaluate a nurse-led, multi-agency drop-in clinic for children and young people with ADHD.	Pre-/post design intervention	Nurse Clinics	6–18 years old (*n = 62)*	Nurse-Led, Multi-Agency Drop-In Clinic	- Significant decrease in the time consultant paediatricians spent on the phone with service users. - Contact with ADHD nurse specialists increased, paediatrician involvement decreased, leading to greater uptake of psychosocial interventions.
Sultan et al. [5]	- To evaluate the effectiveness of shared/collaborative care between mental health-care providers and GPs on the outcomes of children and adolescents with ADHD.	Systematic Review	GP Clinics	N/A	5 Models Reviewed	- Evidence for the effectiveness of shared/collaborative care models showed low levels of meaningful improvement. - The improvement in symptoms was statistically significant, but from a clinical standpoint, it was considered minimally to moderately effective. - GPs comfort of treating ADHD improved. - Increased capacity for diagnosing and managing ADHD did not show improvement.
Shahidullah et al. [12]	-To inform research and practice for primary care behavioural health providers	Systematic Review	GP Clinics	N/A	6 Models Reviewed	- Four of the models demonstrated improvement in accessibility to care. - Results did not indicate that one model was superior to another. - Models ranged from coordinated/telephonic psychiatric services to -Fully integrated services offering approaches that are dependent on the local community needs. - Integration of behavioural health clinician in primary care lessens the issue of poor off-site referral follow through. - Limited scope of studies weakens the ability to draw conclusions and generalisations of these models.

^1^ Attention Deficit Hyperactivity Disorder

^2 ^General Practitioner

^3^ Digital Mental Health Intervention

^4^ Randomized Controlled Test

^5^ Doctor Office Collaborative Care

### Inclusion and exclusion criteria

Eligibility for study inclusion consisted of the following: (1) Peer-reviewed studies reported in English, (2) children or adolescents (age < 18 years old), (3) studies assessing and reporting the cost-effectiveness or efficacy of ADHD care models implementation, (4) study design: randomised trials or non-randomised trials, economic or non-economic studies, observational studies, (5) publication date did not factor into the search strategy. The exclusion criteria consist of the following: (1) did not meet the above criteria, and (2) abstracts, editorials, and opinion pieces.

### Study selection process and data extraction

The search results from each database were exported into Endnote [16], and then imported into a Covidence Systematic Review Software [17]. Two-stage screening process including (1) title and abstract screening, and (2) full-text screening was conducted by two independent reviewers. Disagreements during the screening processes were resolved through discussion until consensus was reached. A data extraction template was developed in Covidence Systematic Review Software. The primary reviewer (RC) completed the data extraction template, which was crossed check by the second reviewer (KM). The extracted information describes the author, aim, population, ADHD status, study design, model description, study measurements, significant results, and key findings.

### Quality assessment

The included studies were assessed for methodological quality by two independent reviewers using the National Heart, Lung, and Blood Institute (NHLBI), which was used for testing and ascertaining any potential flaws in the study methods or implementation (Appendix B, Table B1). In the NHLBI controlled intervention studies, as well as in before-and-after (pre-post) studies without a control group, studies were classified as ‘good,’ ‘fair,’ or ‘poor’ based on the percentage of criteria met: ‘good’ if at least 80% were met, ‘fair’ if 51–71% were met, and ‘poor’ if 50% or fewer were met [18].

## Results

### Study characteristics

**Fig. 1:**
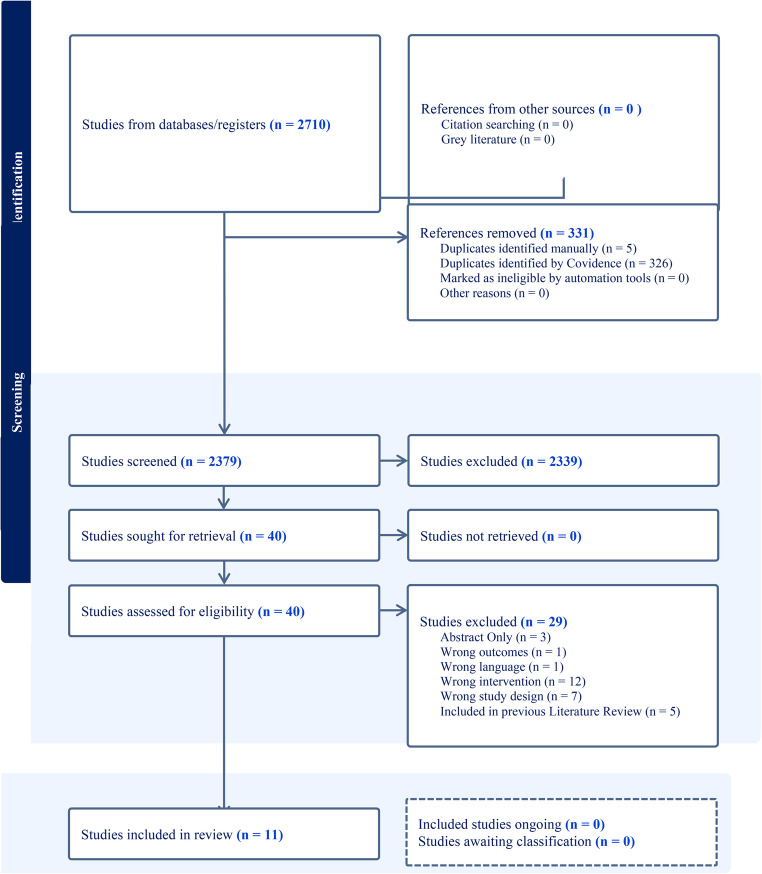
PRISMA Diagram

The search generated 2710 results and was narrowed down to 15 studies (Fig. 1). However, during the full-text screening process it was observed that the inclusion of the systematic review conducted by [12]synthesised 5 selected studies [19–23].

To avoid repetitive summarisation and analysis, the final analysis consisted of 11 articles. The included studies spanned across 3 countries in 3 different continents: (United States [*n* = 9]), (United Kingdom [*n* = 1]), and (United Arab Emirates [*n* = 2]) (Table 1). Of the 11 studies, two used a randomised controlled trial study design [4, 24], four used longitudinal observational cohort design [1, 7, 10, 11], two used retrospective cohort designs [7, 8], two were systematic reviews [5, 12] and one [3] used a pre/post design (Table 1). The quality of 29% of studies (*n* = 3) were rated as “Good”, 63% of studies (*n* = 7) were rated as “Fair”, and the remaining 8% of studies (*n* = 1) were rated as “Poor” (Appendix B, Table 1). The 11 studies, represented 7 ADHD care models:

- ITAs: Integrated Care Arrangements [2, 8, 12].
- DOCC: Doctor Office Collaborative Care [12, 24].
- MAACC: Mood, Anxiety, DHD Collaborative Care Program [10, 11].
- DMHI: Digital Mental Health Intervention [7].
- Nurse-Led, Multi-Agency Drop-In Clinic [3].
- Collaborative Tele-Mental Health Services [4].
- Quality Improvement (QI) Model [1].

### ADHD care models

Almost half of the included peer-reviewed articles [2, 7, 8, 11, 12, 24] have cited the American Academy of Paediatrics (AAP) recommendation of combination therapy using both cognitive behavioural therapy and medication treatment as the benchmark in treating children and adolescents with ADHD. Among the 11 included studies, there are 7 ADHD care models involving a multitude of complexities. Three of the studies suggested [2, 7, 24] adopting a medical family therapy approach, consisting of a care manager or a behavioural health practitioner (BHP) delivering parent behavioural training.

ADHD diagnosis and treatment were provided via tele-health [4] (from GPs in childcare clinics), drop-in clinics [3] (urgent access to ADHD nurse specialists to provide psychosocial interventions), digital mental health interventions [7] (development and coordination of behaviour care treatment plan), Mood Anxiety, ADHD Collaborative Care model [25] (coordination between GPs and child psychotherapist on psychotherapy/pharmacotherapy treatment plan), and fully integrated models (GPs and behavioural health professionals share physical space and medical systems) (Table 1). The Doctor Office Collaborative Care model incorporates ADHD behavioural on-site services that are delivered by both the GPs and care managers[12, 24]. The behavioural on-site services consisted of modules consisted of strategies for goal setting, emotion regulation, and training in parenting or social skills. The Digital Mental Health Intervention model consists of a non-pharmacological approach, where the GP and members of the mental health team develop a cognitive behaviour therapy (CBT) treatment plan and assess the effectiveness through video-based care sessions [7].

### Measuring ADHD

The included research consisted of a multitude of screening toolkits that were used in the assessment and treatment of ADHD in primary care settings. The screening toolkits are a necessity to ensure that children and adolescents are meeting the diagnostic criteria of ADHD set forth by the American Academy of Paediatrics (AAP) [8].The purpose of using these toolkits is not only to obtain evidence of ADHD symptoms, but also to rule out other mental health disorders [2]. This requires the involvement of parents, teachers, GPs, and mental health professionals. The Quality Improvement (QI), Doctor Office Collaborative Care (DOCC), Tele-Mental Care, Mood, Anxiety, DHD Collaborative Care Program (MAACC), and Integrated Arrangement models had parents complete the Vanderbilt Behavioural Assessment Scale [1, 2, 4, 10–12, 24]. The Vanderbilt ADHD rating scales were completed by both parents and teachers during the initial assessment and follow-up in the QI model[1]. Satisfaction questionnaires using a Likert-scale with service were completed by children/adolescents in the nurse-led, multi-agency drop-in clinic and Digital Mental Health Intervention [3, 7]. These toolkits were frequently used at baseline to measure the ADHD symptoms and at follow-ups to evaluate whether there was an improvement in symptoms. Multiple studies have suggested [5, 7, 12] the measuring of ADHD symptoms throughout the intervention determines the level of the models’ effectiveness.

### Effectiveness evaluation

#### Access to care

Three of the seven models reported accessibility to care data. The Doctor Office Collaborative Care (DOCC)which used a psychosocial intervention administered by a behavioural health clinician on-site, was tested in an RCT to extend prior research [24]. The DOCC model resulted in higher rates of treatment initiation (99.4 vs. 54.2%; *p* < 0.001), as well as treatment completion (76.6 vs. 11.6; *p* < 0.001) [24]. The DOCC model was a viable intervention that produced greater rates of services provided for ADHD by GPs or other staff members (95.0% vs. 81.1%, *p* < 0.003) than the control group [24].

Moore et al. [2] and Chen et al. [8] conducted retrospective cohort analyses of integrated care models. Chen et al. [8] reported that children diagnosed by GPs who were both co-located and co-affiliated had a higher treatment rate compared to those diagnosed by co-located but non-co-affiliated GPs (73% vs. 75%; *P* = 0.423). However, the treatment rates were more comparable for those diagnosed by non-co-located general practitioners (GPs), with a higher rate in co-affiliated GPs (82% vs. 73%; *P* = 0.004) [8]. The participants from Moore et al. [2] in the co-located/co-affiliated group were twice as likely to initiate treatment than those who were diagnosed by non-co-located GPs. They were more likely to be engaged with a psychologist (OR −1.44, *P =* 0.048), consult with a psychiatrist (OR −1.18, *P =* 0.036), be referred for behavioural treatment outside the practice (OR −1.58, *P =* 0.027), and have academic reports gathered for follow-up (OR 1.46, *P =* 0.25).

The digital mental health intervention (DMHI) used by Lawrence-Sidebottom et al. [7] was found to be a formable model in the deliverance of ADHD services. A group of participants with at least two assessments, inattention scores improved by 71.0% (*n* = 22), with scores decreasing from an average of 19.65 (moderate symptoms) in the first assessment to 16.13 (mild symptoms) in the final assessment, showing an average decrease of 3.52 points (z = −3.22, *P =* 0.001). For hyperactivity, 60.0% (*n* = 9) of members showed improvement, with scores decreasing by an average of 3.07 points (z = −1.97, *P =* 0.049) [7]. The accessibility of care improved substantially, with children in crisis being assessed within three weeks, compared to the usual wait time of four to six weeks for traditional consultations. This accessibility likely contributed to a marked increase in telephone consultations related to ADHD care (*P* < 0.001), reflecting the system’s effectiveness in meeting the needs of service users [7].

### Children’s outcome from care

The three studies conducted by Epstein et al. [1], Parkhurst et al. [10, 11] reported the effectiveness of the Mood, Anxiety, ADHD Collaborative Care (MAACC) and Quality Improvement (QI) [1, 10, 11] were found to be feasible to improve the accessibility to ADHD services. The MAACC is an extension of the Chronic Care Model (CCM), which was a foundational tool for providing ADHD treatment training for GPs and paediatricians [1].

Parkhurst et al. [10, 11] revealed significant improvements in parent-reported ADHD symptoms, particularly inattention and mixed symptoms (inattention + hyperactivity). Both trials reported significant improvements in inattention; a statistically significant mean difference (Mdiff = 4.69) (*P* = 0.003) Parkhurst et al. [10], and a minor effect size (d = 0.29) Parkhurst et al. [11].

As opposed to Chen et al. [8], Moore et al. [2] did not have a non-co-located cohort. The co-located/co-affiliated cohort from Moore et al. [2] were able to provide more resources and advocacy for special education compared with the co-located site group (24% vs. 11%). Also, the co-located/co-affiliated cohort was found to have more resources on parent education on ADHD (1.18 vs. 0.53 average visits, *t*[1.145.12] = 2.49, *P* = 0.014) [2].

### Key factors influencing the effectiveness of care models

#### Effectiveness barriers

There are numerous factors that lessen the effectiveness of these treatment models. These factors include, but are not limited to, shortages of mental health specialists [1, 8] absent parental engagement [3, 7], lack of paediatric primary care clinician training [11], minimal teacher involvement [24], and understanding of ADHD [2].

### Mental health workers

The included research in this review indicated mental health professionals as integral to collaborative care models [5, 8, 10]. The included research highlights accessibility issues due to a shortage of behavioural health specialists, psychologists, psychiatrists, mental health nurses, and social workers. These accessibility issues extend to not just a shortage of mental health workers, but a lack of training and absence of certified mental health specialists in providing referrals [8]. Consequently, there is less opportunity for general practitioners (GPs) to engage in ADHD treatment training. While Moore et al. [2] and Chen et al. [8] both note that the sharing of physical space between mental health-care providers and GPs are essential and resourceful component of ADHD care models, however, Parkhurst et al. [10] highlights the lack of space in independent practices and inability to cover the costs of the mental health professionals. One of the collaborative care studies [10] points out that urban and suburban practices are more likely to have local hospitals and mental health professionals connections to refer children and adolescents with ADHD for treatment, while rural practices are constrained by limited available resources.

### Race/Ethnicity

The included research [1, 7, 8] demonstrates collaborative care models’ effectiveness cannot be universally implemented due to numerous factors, including covariates and sample size. Doctor Office Collaborative Care and Digital Mental Health Intervention models [7, 8] demonstrated improvement in collaboration but lacked representative samples. While Chen et al. [8] had a more diverse sample size, Caucasian children with ADHD were more likely to receive diagnosis and treatment than Black and Hispanic children, regardless of the care model [8]. Despite higher treatment rates with co-located and co-affiliated GPs, there was no significant benefit for different racial or ethnic groups.

#### Cost-Effectiveness evaluation

Although the included studies in this review did not explore or report cost-effectiveness in the implementation of these collaborative care models, the cost of implementation is a recurring concern and barrier. Despite the collaborative care models demonstrating effectiveness in the reduction of ADHD symptoms, the financial viability of implementing these collaborative care models is either not reported or monitored. While most of the included research acknowledged philanthropic funding was needed for implementation, only two studies highlighted the need to evaluate the financial feasibility of implementing these care models through a longitudinal randomised controlled trial [1, 10]. The objective of tracking costs and reimbursement to assess financial viability is only found in the analysis from one study [10]. The initiation of the Mood, Anxiety, ADHD Collaborative Care model was unable to cover all the programmatic costs, resulting in the necessity for philanthropic support to cover the remaining costs [10]. Sfar-Gandoura et al. [3] encountered a similar issue in their nurse-led, multi-agency drop-in clinic, underscoring the necessity of philanthropic support to cover the psychosocial intervention.

It is important to highlight that 8 of the 11 included articles are from the US. This is notable because the US healthcare system is much different than other countries that have published research on ADHD care models. Although the US is a leader in medical research and technology, the cost of healthcare is very expensive. The annual health system costs per patient varies significantly, with children diagnosed with ADHD experiencing higher expenses ($722-$11,555) compared with those without the condition ($179-$3,646) [26]. The US does not utilise universal healthcare coverage model, which means accessibility to care is highly variable. Accessibility to healthcare in the US may play a larger role, as the included research consistently points to limitations of non-representative sample size and high dropout rates among participants. While there may be a host of other reasons for these limitations, accessibility in the US healthcare system cannot be dismissed. The cost of ADHD treatment has been established to be a barrier for parents or caregivers to seek out ADHD treatment for their children [27]. Therefore, ADHD care models need to be customised and adapted to appropriately address these limitations.

## Discussion

### Summary of findings

The synthesis of the current research in this review highlights a multitude of findings associated with ADHD care models’ collaborative treatment approach and service delivery. Our review found that integrated and collaborative care models are effective in the reduction of ADHD symptoms through comprehensive and holistic treatment. The noticeable gap in the current literature on the cost-effectiveness of ADHD care models amplifies the.

In line with previous systematic review findings [5, 12], we found that general practitioners (GP) were essential in paediatric mental health care, particularly in diagnosing and treating ADHD. The increased comfort level of GPs treating children and adolescents is demonstrated by the decrease in a parent-reported of their child’s ADHD symptoms [11]. As noted in previous systematic reviews [5, 12], there is a necessity for increased education and resources for GPs to improve access to the diagnosis and treatment for children and adolescents with ADHD [10, 11, 24]. The integration of ADHD treatment training into primary care can improve ADHD treatment outcomes by addressing common challenges like poor referral follow-through, educational barriers, and time constraints faced by GPs [5, 12]. This integration allows for more efficient and effective management of ADHD, delivering results comparable to, or even exceeding, traditional treatment methods, while reducing the burden on GPs.

The included research places much emphasis on how collaborative and integrated care are instrumental in the improvement and reduction of ADHD symptoms. Collective cooperation within the healthcare system can range from minimal interaction, such as when GPs refer patients to behavioural health practitioners with little communication, to highly integrated models where GPs and mental health providers share physical space, scheduling, patient charts, and even care responsibilities [2, 8]. Due to ADHD and other complex co-morbidities requiring a multi-disciplinarian approach, ADHD care models, where GPs work alongside mental health professionals (such as behaviour health practitioners, nurse specialists, community therapists, coaches, psychologists, social workers or psychiatrists), are particularly beneficial [1–3, 8, 11, 12]. These collaborative and integrated models ensure patients receive holistic care tailored to their needs.

Our review provides limited information on the cost-effectiveness of implementing ADHD collaborative care models. The cost of treating ADHD is substantial (e.g. [26] annual total costs per individual ranged from US$831 to US$20,538). Children with ADHD incur higher medical expenses compared to their peers without the condition, and these increased costs persist into adulthood [26, 28–30]. Therefore, it is critical to assess the costs and cost-effectiveness of these innovative ADHD care models to better implement and sustain integrated/collaborative care models for ADHD. Our review also highlights a gap on the costs of educational resources for children and adolescents with ADHD. High medical costs for ADHD treatment may be indicative of a greater need for resource allocation in other areas of ADHD treatment such as special education or cognitive behaviour therapy.

Increased accessibility of ADHD treatment when utilising tele-health suggests that telehealth could be a potential service for patients located in rural areas with limited resources [4]. Included studies in our review shows that when psychopharmacologic treatment was prescribed, this has led to significant reduction in inattention and hyperactivity symptoms [7, 10].

#### Policy implications and future research direction

While both systematic reviews [5, 12] concur that collaborative and integrated care models are ideal for ADHD treatment, however, there is a lack of focused research on its effectiveness and cost-effectiveness. The limited evidence found in our review suggests that ADHD patients involved in collaborative care programs experience significant reductions in symptoms such as inattention and hyperactivity. Future research implementing and evaluating the cost/effectiveness of these collaborated or integrated care models would be needed.

We found that key factors contributing to the success of integrated care include having sufficient staffing, availability of appointment slots, and dedicated nonclinical time for provider collaboration. In these settings, behaviour health practitioners may also adopt frameworks such as medical family therapy to further enhance integration [7]. This review found that many ADHD care models incorporate measurement-based care for monitoring symptoms over an extended period of time [1–3, 5, 10, 11]. The implementation of ADHD care models has financial consequences threatening the feasibility of facilitating the intervention. The scarce research on ADHD care models could be due to the likelihood of the financial burden. Future implementation of these care models would need to consider these relevant factors to ensure the ADHD care models’ feasibility including the financial viability of these care model.

The two previous reviews [5, 12] indicate that teachers are integral in the diagnosis of ADHD due to the symptoms needing to be present in multiple in settings. While the included research emphasises general practitioner ADHD treatment training as a key component to the success of ADHD care models, schoolteachers are rarely mentioned beyond their participation in completing the symptom rating scale. Much of the included research acknowledges the challenges children with ADHD can encounter in the education setting. The knowledge and ability to effectively teach students and adolescents with ADHD could potentially be a determining factor for the effectiveness of ADHD care models. While there is very little evidence in ADHD training for schoolteachers [31], there has been promising evidence indicating that the implementation of parent training curriculum has benefited both parents and children’s outcomes. Meta-analyses of randomised controlled trials indicate that parent training enhances parent-child relationships and reduces challenging behaviours in children with ADHD, as reported by both parents and independent observers who are unaware of the treatment assignment [32–34]. Furthermore, the success of general practitioner ADHD treatment training has the potential to set a roadmap to construct and design teacher training curriculum that encompasses compassionate and resourceful support for teachers to provide the necessary support for children and adolescents to achieve academic success.

There is a gap in the research on whether these collaborative care models are financially sustainable in maintaining if they are heavily reliant on philanthropic funding. Further research on collaborative care models should include an objective of identifying if the collaborative care models can be sustainable without philanthropic funding. Implementation of ADHD care models has financial consequences threatening the feasibility of facilitating the intervention. Governments can play a crucial role in aiding and facilitating the implementation of ADHD care models. An increase in funding towards mental health services has reflected an increase in cost-effective strategy aimed at improving accessibility to ADHD health services [35]. Government grants can particularly be beneficial in this area. For example, earlier this year, the Centers for Disease Control and Prevention submitted a grant application to the US government Department of Health and Human Services [35]. The aim is to improve accessibility to evidence-based services for individuals with Tourette Syndrome or ADHD and their families, ensuring outreach to diverse communities [35]. It also seeks to enhance healthcare professionals’ ability to diagnose and treat these conditions and support education professionals in implementing strategies that aid academic success for affected children. Policy changes to national service delivery systems should focus on strategies to enhance both the physical and financial accessibility of healthcare providers who are trained to diagnose and treat ADHD in children, with the aim of reducing waiting times and costs associated with accessing and utilizing services.

While fully replicating these models may not be feasible, implementing specific elements—such as training GPs in ADHD treatment and utilising telehealth—could be viable. Incorporating telephone consultations into the model could also reduce the need for full psychiatric assessments, making care more accessible. This would be particularly beneficial for rural community-based clinics where access to behavioural health service is burdened and inundated with requests for ADHD treatment [12, 36]. Investing in these studies is essential, as they have the potential to build sustainable capacity and improve long-term care delivery.

#### Strengths and limitations

Our study’s strength includes the comprehensive search for the most recent current literature on collaborative care models in children and adolescents with ADHD. As alluded to earlier, a notable limitation of this research paper was the limited amount of research found evaluating the effectiveness and cost-effectiveness of ADHD care models and the limited research conducted in countries outside of the US. While two studies [3, 4] were conducted in the UK and UAE, respectively, the included research is predominately from the US. In comparison with other industrialised countries—United Kingdom, United Arab Emirates, Australia, New Zealand, Canada, and Germany—the US does not operate a universal health coverage model [37]. The absence of universal health coverage in the US perpetuates accessibility and affordability disparities to receiving quality treatment [37]. As a result, the issue of accessibility to ADHD diagnosis and treatment may not be an issue for countries that do use a universal health coverage model. This limits our ability to fully explore and understand the implementation of ADHD care models in other countries with different healthcare systems. The scarce literature exploring the effectiveness and cost-effectiveness of ADHD care models and the lack of a comparison group in most studies limit our ability to determine whether improvements are due to the care model or other factors, limiting confidence in the current evidence for policy and practice. Thus, caution should be taken in interpreting our study findings. The exclusion of non-English languages may have resulted in excluding valuable research in this review.

## Conclusions

This review found seven ADHD care models that were implemented to diagnose and treat ADHD among children and adolescents. These care models showed an improvement in service delivery, including a reduction in ADHD symptoms but no cost-effectiveness has been explored in the current literature. Future research on ADHD care models for managing ADHD in children and adolescents should aim to enhance the efficiency and practicality of these approaches to maximise their cost-effectiveness and effectiveness. Key efforts should focus on identifying the most beneficial components of collaborative care that can help realign ADHD management with evidence-based practices. The limited research in this area highlights the necessity to conduct further research exploring the effectiveness and cost-effectiveness of ADHD care models. Future research should explore the financial sustainability of ADHD care models.

## Data Availability

No datasets were generated or analysed during the current study.

## Appendix A

**Table 2:** Search terms used for each database

1	TI OR AB	“cognitive behavior” OR “cognitive behaviour” OR “ADHD” OR “ADD” OR “attention deficit disorder with hyperactivity” OR “mental health disorder” OR “pervasive developmental disorder” OR “neurodevelopmental disorder”	Search with AND
2	TI OR AB	“integrated care models” OR “treatment guidelines” OR “multidisciplinary model” OR “collaborative care” OR “care programs” OR “health models” OR “shared care”	Search with AND
3	TI OR AB	“effect*” OR “impact*” OR “efficacy*” OR “evaluat* OR “impression*” OR “use” OR “productiv*” OR “use*” OR “capability*” OR “sufficiency*” OR “influence” OR “value*” OR “compet*” OR “validity*” OR “cost-effectiveness analys*” OR “cost effective*” OR “cost-utility” OR “cost utility*” OR “return on investment*” OR “cost benefit*” OR “measures of effectiveness” OR “cost-benefit analys*” OR “resource allocation*” OR “economic analys*” OR “cost-minimi*” OR “economic evaluation framework” OR	Search with AND

## Appendix B

**Table 3:** Quality assessment using the national heart, lung, and blood institute

Quality Assessment Tool for Before-After (Pre-Post) Studies with No Control Group	Sfar-Gandoura et al., (2017)
Was the study question or objective clearly stated?	Y
Were eligibility/selection criteria for the study population prespecified and clearly described?	Y
Were the participants in the study representative of those who would be eligible for the test/service/intervention in the general or clinical population of interest?	Y
Were all eligible participants that met the prespecified entry criteria enrolled?	N
Was the sample size sufficiently large to provide confidence in the findings?	Y
Was the test/service/intervention clearly described and delivered consistently across the study population?	N
Were the outcome measures prespecified, clearly defined, valid, reliable, and assessed consistently across all study participants?	Y
Were the people assessing the outcomes blinded to the participants' exposures/interventions?	N
Was the loss to follow-up after baseline 20% or less? Were those lost to follow-up accounted for in the analysis?	NR
Did the statistical methods examine changes in outcome measures from before to after the intervention? Were statistical tests done that provided p values for the pre-to-post changes?	Y
Were outcome measures of interest taken multiple times before the intervention and multiple times after the intervention (i.e., did they use an interrupted time-series design)?	Y
If the intervention was conducted at a group level (e.g., a whole hospital, a community, etc.) did the statistical analysis consider the use of individual-level data to determine effects at the group level?	N

*Y, yes; N, no; CD, cannot determine; NA, not applicable; NR, not reported; N/A, non-applicable

### Quality rating: Poor

While the study has some strengths, such as a well-defined question, adequate eligibility criteria, a representative sample, trustworthy end measures, and a strong statistical technique, there are several areas that require improvement. These include the failure to enroll all eligible individuals, insufficient clarity and consistency in the intervention description, and the absence of blinding for outcome assessors. Furthermore, there is uncertainty concerning the loss of follow-up and whether it was adequately addressed in the analysis. Failure to account for individual-level data in group-level treatments may potentially jeopardize the study. Addressing these shortcomings would increase the overall validity and dependability of the findings.

**Table 4:** These systematic reviews demonstrate several strengths, including a well-defined research question, clearly established eligibility criteria, a thorough search strategy, and transparent reporting of study characteristics. However, there are key areas for improvement. Specifically, it would be beneficial to ensure that study inclusion and exclusion are conducted independently by multiple reviewers, and that the quality of the studies is consistently assessed to minimize bias and enhance the reliability of the findings. Additionally, these reviews would benefit from assessing publication bias to ensure it includes all relevant studies, not just those with positive or significant results. These improvements would strengthen the credibility and comprehensiveness of the reviews conclusions

Quality Assessment for Systematic Reviews	Shadidullah et al., (2018)	Sultan et al., (2018)
Is the review based on a focused question that is adequately formulated and described?	Y	Y
Were eligibility criteria for included and excluded studies predefined and specified?	Y	Y
Did the literature search strategy use a comprehensive, systematic approach?	Y	Y
Were titles, abstracts, and full-text articles dually and independently reviewed for inclusion and exclusion to minimize bias?	CD	CD
Was the quality of each included study rated independently by two or more reviewers using a standard method to appraise its internal validity?	CD	CD
Were the included studies listed along with important characteristics and results of each study?	Y	Y
Was publication bias assessed?	N	N
Was heterogeneity assessed?	N/A	N/A
Is the review based on a focused question that is adequately formulated and described?	Y	Y
Were eligibility criteria for included and excluded studies predefined and specified?	Y	Y
Did the literature search strategy use a comprehensive, systematic approach?	Y	Y
Were titles, abstracts, and full-text articles dually and independently reviewed for inclusion and exclusion to minimize bias?	CD	CD
Was the quality of each included study rated independently by two or more reviewers using a standard method to appraise its internal validity?	CD	CD
Were the included studies listed along with important characteristics and results of each study?	Y	Y
Was publication bias assessed?	N	N
Was heterogeneity assessed	N/A	N/A
	Quality Rating: Good	Quality Rating: Fair

**Table 5:** The studies present several strengths, including clearly defined research question, a well-characterized study population, and a sufficient timeframe to observe potential associations. However, there are notable limitations that need to be addressed. Key details about the validity and consistency of exposure measures are lacking, and it is unclear whether outcome assessors were blinded, which could introduce bias. Furthermore, the absence of sample size justification, inclusion/exclusion criteria details, and adjustments for confounding variables weakens the overall reliability of the findings. The lack of information on loss to follow-up is another significant concern, as it raises the possibility of bias affecting the results

Quality Assessment for Observational Cohort and Cross-Sectional	Lawrence-Sidebottom et al., (2023)	Moore et al., (2017)
Was the research question or objective in this paper clearly stated?	Y	Y
Was the study population clearly specified and defined?	Y	Y
Was the participation rate of eligible persons at least 50%?	N/A	N
Were all the subjects selected or recruited from the same or similar populations (including the same time period)?	Y	N
Were inclusion and exclusion criteria for being in the study prespecified and applied uniformly to all participants?	Y	N
Was a sample size justification, power description, or variance and effect estimates provided?	N	Y
For the analyses in this paper, were the exposure(s) of interest measured prior to the outcome(s) being measured?	Y	CD
Was the timeframe sufficient so that one could reasonably expect to see an association between exposure and outcome if it existed?	Y	CD
For exposures that can vary in amount or level, did the study examine different levels of the exposure as related to the outcome (e.g., categories of exposure, or exposure measured as continuous variable)?	Y	CD
Were the exposure measures (independent variables) clearly defined, valid, reliable, and implemented consistently across all study participants?	CD	CD
Was the exposure(s) assessed more than once over time?	Y	N/A
Were the outcome measures (dependent variables) clearly defined, valid, reliable, and implemented consistently across all study participants?	Y	Y
Were the outcome assessors blinded to the exposure status of participants?	CD	CD
Was loss to follow-up after baseline 20% or less?	N	N/A
Were key potential confounding variables measured and adjusted statistically for their impact on the relationship between exposure(s) and outcome(s)?	N	N/A
	Quality Rating: Fair	Quality Rating: Fair

**Table 6:** There are several areas of concern that could affect the validity of these studies’ findings. The lack of information on participation rates and the absence of sample size justification in these studies raises questions about the studies’ power and the risk of selection bias. Furthermore, while the studies use repeated exposure assessments, it does not provide sufficient detail on whether confounding variables were adequately controlled, which could distort the observed relationships. Additionally, potential biases from the blinding of participants and providers remain unclear. The absence of details about loss to follow-up also creates concerns regarding incomplete data and the potential for bias

Quality Assessment for Observational Cohort and Cross-Sectional	Parkhurst et al., (2022)	Parkhurst et al., (2023)	Chen et al., (2021)
Was the research question or objective in this paper clearly stated?	Y	Y	Y
Was the study population clearly specified and defined?	Y	Y	Y
Was the participation rate of eligible persons at least 50%?	N	CD	CD
Were all the subjects selected or recruited from the same or similar populations (including the same time period)?	Y	Y	N
Were inclusion and exclusion criteria for being in the study prespecified and applied uniformly to all participants?	Y	Y	Y
Was a sample size justification, power description, or variance and effect estimates provided?	N	N	Y
For the analyses in this paper, were the exposure(s) of interest measured prior to the outcome(s) being measured?	Y	Y	Y
Was the timeframe sufficient so that one could reasonably expect to see an association between exposure and outcome if it existed?	Y	Y	CD
For exposures that can vary in amount or level, did the study examine different levels of the exposure as related to the outcome (e.g., categories of exposure, or exposure measured as continuous variable)?	Y	Y	Y
Were the exposure measures (independent variables) clearly defined, valid, reliable, and implemented consistently across all study participants?	Y	Y	Y
Was the exposure(s) assessed more than once over time?	Y	Y	N
Were the outcome measures (dependent variables) clearly defined, valid, reliable, and implemented consistently across all study participants?	Y	Y	Y
Were the outcome assessors blinded to the exposure status of participants?	CD	CD	CD
Was loss to follow-up after baseline 20% or less?	N	CD	CD
Were key potential confounding variables measured and adjusted statistically for their impact on the relationship between exposure(s) and outcome(s)?	CD	N	N
	Quality Rating: Good	Quality Rating: Fair	Quality Rating: Fair

**Table 7:** The design of the study has been carried out effectively for the most part. It features a clearly articulated aim, predetermined eligibility criteria, and an intervention that was delivered consistently. The measures of outcome were well-defined and assessed reliably. Nevertheless, there are weaknesses. The sample size was not adequate to ensure confidence in the findings. The study does not clarify whether the participants were representative of the wider population that would generally use the test or intervention

Quality Assessment Before-After (Pre-Post) Studies With No Control Group	Epstein et al. (2010)
Was the study question or objective clearly stated?	Y
Were eligibility/selection criteria for the study population prespecified and clearly described?	Y
Were the participants in the study representative of those who would be eligible for the test/service/intervention in the general or clinical population of interest?	CD
Were all eligible participants that met the prespecified entry criteria enrolled?	Y
Was the sample size sufficiently large to provide confidence in the findings?	N
Was the test/service/intervention clearly described and delivered consistently across the study population?	Y
Were the outcome measures prespecified, clearly defined, valid, reliable, and assessed consistently across all study participants?	Y
Were the people assessing the outcomes blinded to the participants' exposures/interventions?	CD
Was the loss to follow-up after baseline 20% or less? Were those lost to follow-up accounted for in the analysis?	CD
Did the statistical methods examine changes in outcome measures from before to after the intervention? Were statistical tests done that provided p values for the pre-to-post changes?	Y
Were outcome measures of interest taken multiple times before the intervention and multiple times after the intervention (i.e., did they use an interrupted time-series design)?	Y
If the intervention was conducted at a group level (e.g., a whole hospital, a community, etc.) did the statistical analysis consider the use of individual-level data to determine effects at the group level?	CD
	Quality Rating: Good
